# Ferroptosis and Its Role in Chronic Diseases

**DOI:** 10.3390/cells11132040

**Published:** 2022-06-27

**Authors:** Wenli Hu, Kehong Liang, Hong Zhu, Chong Zhao, Hongbo Hu, Shutao Yin

**Affiliations:** 1 College of Food Science and Nutritional Engineering, China Agricultural University, Beijing 100083, China; sy20183061027@cau.edu.cn (W.H.); zhaoch0206@cau.edu.cn (C.Z.); hongbo@cau.edu.cn (H.H.); 2 Institute of Food and Nutrition Development, Ministry of Agriculture and Rural Affairs, Beijing 100083, China; liangkehong@caas.cn (K.L.); zhuhong@caas.cn (H.Z.)

**Keywords:** ferroptosis, basic characteristics of ferroptosis, regulation of ferroptosis, chronic diseases, food-borne active ingredients

## Abstract

Ferroptosis, which has been widely associated with many diseases, is an iron-dependent regulated cell death characterized by intracellular lipid peroxide accumulation. It exhibits morphological, biochemical, and genetic characteristics that are unique in comparison to other types of cell death. The course of ferroptosis can be accurately regulated by the metabolism of iron, lipids, amino acids, and various signal pathways. In this review, we summarize the basic characteristics of ferroptosis, its regulation, as well as the relationship between ferroptosis and chronic diseases such as cancer, nervous system diseases, metabolic diseases, and inflammatory bowel diseases. Finally, we describe the regulatory effects of food-borne active ingredients on ferroptosis.

## 1. Introduction

The term ‘ferroptosis’, first proposed in 2012, refers to a programmed cell death resulting from iron-dependent lipid peroxidation accumulation. Ferroptosis is distinct from other previously established regulated cell deaths and has specific morphological, biochemical, and genetic characteristics. The regulation of ferroptosis is associated with multiple signal pathways, and there is increasing evidence to suggest its participation in the regulation of numerous diseases. In this paper, we describe the basic features and the regulation mechanisms of ferroptosis, and analyze the application of ferroptosis in chronic diseases such as cancer, nervous system diseases, metabolic diseases, and inflammatory bowel diseases. We also describe the regulatory effects of food-borne active ingredients on ferroptosis.

## 2. Basic Characteristics of Ferroptosis

### 2.1. Morphological Features

Ferroptosis has morphological, biochemical, and genetic features that are unique in comparison with apoptosis, autophagy, necroptosis, and pyroptosis (Table 1) [1,2,3]. Cells that undergo ferroptosis generally show a necrosis-like morphological transformation, including cell membrane rupture, cytoplasmic swelling, and moderate chromatin condensation [4]. At the ultrastructural level, ferroptotic cells usually present mitochondrial shrinkage, an increase in membrane density, reduced or absent cristae, and rupturing of the outer membrane [2]. Since autophagy promotes ferroptosis, autophagy-related ultrastructures, such as double-membrane autophagosomes and various lysosome-related vesicles, are often seen in ferroptotic cells or tissue [5].

### 2.2. Biochemical Features

The two main biochemical features of ferroptosis are iron accumulation and lipid peroxidation. Intracellular free ferrous iron accumulation generates excessive reactive oxygen species (ROS) directly via the Fenton reaction, thereby triggering lipid peroxidation and ferroptosis. Erastin induces ferroptosis by decreasing ferroportin (FPN) and increasing the level of ferrous ions (Fe^2+^) in endometriosis [6]. ROS-mediated autophagy results in ferroptosis via the degradation of ferritin and inducement of transferrin receptor (TFR1) expression to increase intracellular iron levels [7]. Iron overload in the motor cortex has been reported to trigger the ferroptosis of neuronal cells after spinal cord injury [8]. Ferroptosis inhibition attenuates atherosclerosis via the alleviation of lipid peroxidation [9].

### 2.3. Genetic Features

Some genes are overexpressed in ferroptosis. For example, prostaglandin-endoperoxide synthase 2 (PTGS2/COX2) is known to play an important role in prostaglandin biosynthesis [10], while Acyl-CoA synthetase long-chain family member 4 (ACSL4) is an enzyme that increases the polyunsaturated fatty acid (PUFA) content in phospholipids, making them more sensitive to ferroptosis [11]. Other genes, such as glutathione peroxidase 4 (GPX4) and ferritin heavy chain 1 (FTH1), are downregulated in ferroptosis. GPX4 converts the cytotoxic lipid peroxides (L-OOH) into the corresponding alcohols (L-OH) under glutathione (GSH), and the suppression of GPX4 activity leads to lipid peroxides accumulation and ferroptosis. FTH1 is an iron-storage protein and, therefore, the reduction of its expression facilitates iron accumulation and ferroptosis.

## 3. Regulation of Ferroptosis

**Figure 1:**
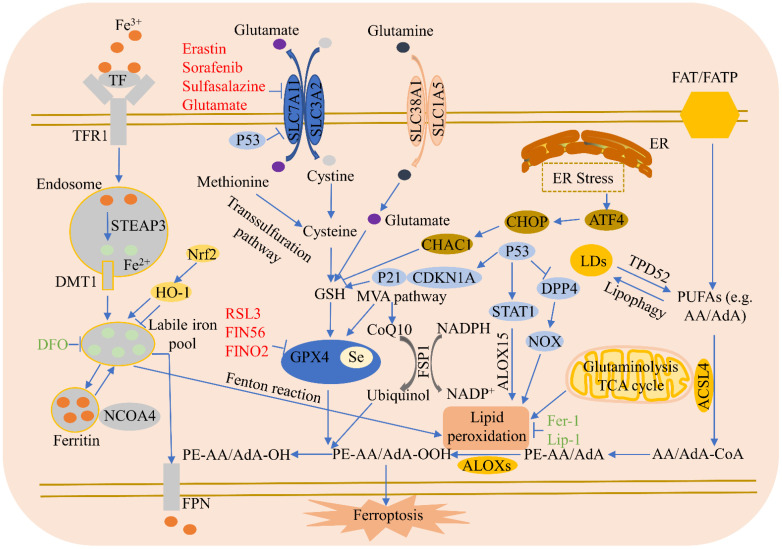
Ferroptosis regulatory pathways. Ferroptosis regulatory pathways can be roughly classified into three types: The first involve iron metabolism, including the nuclear receptor coactivator 4 (NCOA4) regulation of ferritin degradation, and the Nrf2-HO-1 pathway, which affects iron. The second is the GSH/GPX4 pathway, including system Xc- inhibition, the transsulfuration pathway, mevalonate pathway (MVA pathway), glutamine pathway, and p53. The third type is that of lipid metabolism, including ACSL4, P53/SAT1/ALOX15, TPD52, and lipophagy, which are related to lipid regulation and ferroptosis, as well as the FSP1-CoQ10-NAD(P)H pathway synergies with GPX4 and GSH, which reduce phospholipid peroxidation and ferroptosis. In addition, endoplasmic reticulum (ER) stress facilitates ferroptosis via ATF4-induced CHAC 1 expression.

Ferroptosis regulatory pathways are shown in Figure 1.

### 3.1. Iron Metabolism

Iron in food is principally Fe^3+^, which combines with transferrin (TF) in serum and is then identified by TFR1 in the cell membrane. Once absorbed by TFR1, Fe^3+^ is reduced to Fe^2+^ through the STEAP3 metalloreductase in the endosome, which is subsequently released from the endosome into the cytosol via solute carrier family 11 member 2 (SLC11A2/DMT1) [4]. The iron-storage protein ferritin plays an antiferroptosis role, including those of ferritin light chain and FTH1, and lysosomes can degrade ferritin to increase free iron levels. NCOA4-mediated ferritinophagy increases the degradation of ferritin by lysosomes, reduces iron storage, and promotes ferroptosis [12]. Finally, the iron-efflux protein FPN exports iron out of cells. Blocking the iron release pathway in cell membranes increases sensitivity to ferroptosis.

### 3.2. Lipid Metabolism

The absorption of free PUFAs such as arachidonic acid (AA) or adrenoxyl (AdA) is mediated by fatty acid translocase (FAT) and fatty acid transport protein (FATP) [13]. ACSL4 facilitates the incorporation of PUFAs into cell membranes and ferroptosis. Lipoxygenases (ALOXs) mediate lipid peroxidation and cause ferroptosis. Tumor protein D52 (TPD52)-mediated lipid storage represses RSL3-induced lipid peroxidation and succedent ferroptosis [14]. Lipophagy, a process of autophagy, degrades lipid droplets (LDs), enhances the production of free fatty acids, and increases lipid peroxidation and ferroptosis [14]. Sterol carrier protein 2 promotes the transportation of peroxidized lipids to mitochondria and accelerates GPX4 depletion-mediated ferroptosis. Finally, acetyl-CoA carboxylase alpha is involved in fatty acid β-oxidation and biosynthesis, and promotes ferroptosis.

### 3.3. Amino Acid Metabolism

Amino acid metabolism is closely related to ferroptosis regulation. Cysteine availability restricts GSH biosynthesis, while cysteine starvation induces GSH depletion and ferroptosis. When the available cysteine is limited, some cells utilize the transsulfuration pathway to transform methionine to cysteine [15]. These cells do not require the cystine/glutamate antiporter system Xc- to import cystine and, therefore, are resistant to system Xc- inhibitor-induced ferroptosis. Glutamate is also an important regulator of ferroptosis. In high concentrations, it suppresses system Xc- and triggers ferroptosis. Glutamine degradation (via glutaminolysis) fuels the tricarboxylic acid (TCA) cycle and provides the basis for necessary biosynthetic processes, such as lipid biosynthesis. Cystine starvation and blocked cystine uptake cannot increase ROS accumulation, lipid peroxidation, and ferroptosis in the presence of glutamine deficiency or the inhibition of glutaminolysis [16].

### 3.4. System Xc-

The amino acid antiporter system Xc- consists of two subunits: SLC7A11 and SLC3A2. System Xc- imports extracellular cystine into cells by exchanging intracellular glutamate. Erastin, sorafenib, sulfasalazine, and glutamate all inhibit SLC7A11 expression, causing cysteine deprivation, GSH deletion, and GPX4 inactivation to trigger ferroptosis [17]. ATF3 enhances the ferroptosis induced by erastin via the repression of system Xc- [18], while AMPK-mediated BECN1 phosphorylation increases ferroptosis by directly inhibiting system Xc- activity [19]. Radiotherapy and immunotherapy enhance lipid oxidation and the ferroptosis of tumor cells by synergistically suppressing SLC7A11 [20]. Sorafenib inhibits system Xc- function and induces ferroptosis [21], while GDF15 knockdown facilitates the ferroptosis induced by erastin via the attenuation of SLC7A11 expression [22]. Cardiac ferritin H deficiency reduces SLC7A11 expression and facilitates ferroptosis and cardiomyopathy [23]. Nrf2 suppresses ferroptosis and reduces intestinal ischemia/reperfusion-induced acute lung injury (IIR-ALI) by increasing SLC7A11 and HO-1 [24]. PARP inhibition increases ferroptosis by inhibiting SLC7A11 and cooperates with ferroptosis inducers in BRCA-proficient ovarian cancer [25], while Nrf2 and STAT3 reduce ferroptosis by regulating SLC7A11, which alleviates IIR-ALI [26]. 

### 3.5. GPX4

RSL3 represses GPX4 activity via covalent bonding with GPX4, resulting in the accumulation of lipid peroxides and, ultimately, ferroptosis. GSH is a co-factor in the catalysis of peroxides by GPX4 to produce alcohols; however, cysteine deprivation leads to GSH depletion, which directly inactivates GPX4, resulting in the subsequent induction of ferroptosis. Legumain has been shown to facilitate tubular ferroptosis via the promotion of chaperone-mediated GPX4 autophagy in acute kidney injury [27], while the selenium-GPX4 axis reduces the ferroptosis of follicular helper T cells [28]. Selenium is necessary for GPX4 to suppress hydroperoxide-induced ferroptosis [29,30]. FINO_2_ promotes ferroptosis through GPX4 inactivation and iron oxidation [31]. The downregulation of GPX4 during myocardial infarction triggers ferroptosis in cardiomyocytes [32]. Dihydroartemisinin (DHA) induces ferroptosis in glioblastoma through the inhibition of GPX4 [33]. SIRT3-activated autophagy promotes ferroptosis by increasing the AMPK/mTOR pathway and reducing GPX4 levels [34]. GPX4 maintains Treg cell activation and reduces antitumor immunity by inhibiting lipid peroxidation and ferroptosis [35]. Kaempferol protects from oxygen-glucose deprivation/reoxygenation-induced neuronal ferroptosis via the upregulation of the Nrf2/SLC7A11/GPX4 axis [36]. Finally, the inactivation of GPX4 was found to trigger ferroptosis and acute renal failure in mice [37].

### 3.6. Ferroptosis Suppressor Protein 1 (FSP1)

FSP1 suppresses ferroptosis independent of GSH. Under nicotinamide adenine dinucleotide phosphate (NADPH), FSP1 reduces ubiquinone, also called coenzyme Q10 (CoQ10), to ubiquinol, which can reduce lipid peroxidation and succedent ferroptosis. The FSP1-CoQ10-NAD(P)H pathway has been shown to synergize with GPX4 and GSH to repress phospholipid peroxidation and ferroptosis [38], while a small molecule, NPD4928, reportedly enhances ferroptosis via the inhibition of FSP1 [39]. Plasma-activated medium promotes ferroptosis by decreasing FSP1 expression in human lung cancer cells [40]. Mesenchymal stem cell derived exosomes inhibit neuron ferroptosis by lncGm36569/microRNA (miR)-5627-5p/FSP1 axis in acute spinal cord injury [41], and MiR-672-3p inhibition facilitates neural recovery via the suppression of ferroptosis by FSP1 [42].

### 3.7. P53

P53 is a tumor-suppressor gene that plays a dual role in ferroptosis, regulating it via either a transcriptional or post-translational mechanism. P53 decreases cystine absorption by transcriptionally suppressing SLC7A11 expression, reduces intracellular GSH, and induces ferroptosis in tumor cells [43]. P53 also enhances ferroptosis via the transcriptional induction of SAT1 or GSL2 [44]. On the other hand, p53 inhibits ferroptosis by transcriptionally inducing CDKN1A/p21 (cyclin-dependent kinase inhibitor 1 A) expression [45]. P53 also limits erastin-induced ferroptosis by blocking dipeptidyl peptidase 4 (DPP4) activity via a transcription-independent mechanism [46].

### 3.8. Heme Oxygenase-1 (HO-1)

HO-1 plays a dual role in ferroptosis induction, associated with both the environment and cell type. HO-1 promotes the ferroptosis induced by erastin in HT-1080 fibrosarcoma cells [47], while tagitinin C promotes colorectal cancer cell ferroptosis by activating the PERK-Nrf2-HO-1 signaling pathway [48]. Ferroptosis inhibition effectively reduces dextran sulfate sodium (DSS)-induced ulcerative colitis, related to Nrf2/HO-1 signaling pathway blocking [49]. However, HO-1 also functions as a negative regulator of ferroptosis, and cetuximab enhances RSL3-induced ferroptosis in KRAS mutant colorectal cancer cells through the inhibition of the Nrf2/HO-1 pathway [50]. Gastrodin alleviates the ferroptosis of HT-22 cells triggered by glutamate by up-regulating the Nrf2/HO-1 signaling pathway [51]. HO-1 plays a key antiferroptotic role in renal epithelial cells [52]. Fraxetin decreases myocardial-infarction-mediated ferroptosis through AKT/Nrf2/HO-1 pathway activation [53]. MiR-3587 inhibitor promotes HO-1 up-regulation, thereby protecting renal tissues from ischemia/reperfusion-induced ferroptosis [54]. Melatonin reduces the level of ferroptosis by activating the Nrf2/HO-1signaling pathway in type 2 diabetic osteoporosis [55]. Panaxydol ameliorates lipopolysaccharide (LPS)-induced acute lung injury via reducing ferroptosis by upregulating the Keap1-Nrf2/HO-1 pathway [56]. Ginkgetin, which is derived from *Ginkgo biloba* leaves, increases cisplatin-induced ferroptosis via the disruption of the Nrf2/HO-1 axis [57].

### 3.9. NCOA4

Recent studies have shown that ferritinophagy, a selective autophagy, can degrade ferritin. NCOA4, a selective cargo receptor, transports ferritin to lysosomes where it is then degraded, then labile iron is released and oxygen radicals are increased to induce ferroptosis [12]. NCOA4-mediated ferritinophagy enhances erastin-induced ferroptosis in HeLa cells [58]. The loss of coatomer protein complex subunit zeta 1 increases NCOA4 expression and the ferroptosis of glioblastoma cell lines [59]. DNA (cytosine-5)-methyltransferase 1 inhibition reduces ferroptosis via NCOA4-mediated ferritinophagy during diabetes myocardial ischemia/reperfusion injury [60]. Formosanin C promotes the ferroptosis of human hepatocellular carcinoma cells by elevating NCOA4-mediated ferritinophagy [61]. Disrupting NCOA4-FTH1 interaction inhibits ferroptosis [62]. NCOA4-mediated ferritinophagy increases the ferroptosis induced by cerebral ischemia [63].

### 3.10. Endoplasmic Reticulum (ER) Stress

ER stress is triggered under different pathological conditions and is closely associated with the course of cell death. Erastin can induce ER stress and up-regulates ER-stress-responsive genes [21]. The eif2α-ATF4 axis is the ferroptotic reagent-activated primary signaling pathway. CHAC 1 is downstream of ATF4 and degrades GSH while promoting the succedent ferroptosis [64].

### 3.11. Common Inducers and Inhibitors of Ferroptosis

The four known mechanisms of ferroptosis inducers are: (1) the inhibition of system Xc- and depletion of intracellular GSH by, for example, erastin, sorafenib, sulfasalazine, or glutamate; (2) the inhibition of GPX4 by, for example, RSL3, which covalently combines with GPX4 to reduce its activity, resulting in the accumulation of toxic lipid peroxides and, ultimately, ferroptosis; (3) the degradation of GPX4 and exhaustion of antioxidant CoQ10 by, for example, FIN56; and (4) the direct oxidation of ferrous iron and lipidome, or indirect inactivation of GPX4 by, for example, FINO_2_ [31]. There are two mechanisms of ferroptosis inhibitors: (1) inhibiting the accumulation of iron, such as that of deferoxamine (DFO), which chelates iron and limits the Fenton reaction to prevent lipid peroxidation; and (2) the inhibition of lipid peroxidation by, for example, ferrostatin-1 (Fer-1), liproxststatin-1 (Lip-1), and vitamin E, which act as radical scavengers to reduce lipid peroxides and effectively block ferroptosis. DFO is commercially available as Desferal, an iron chelator clinically approved for the treatment of acute iron intoxication and chronic iron overload [65]. For the action mechanism, Desferal is a hexadentate molecule that is able to bind free plasma iron and excess iron within cells at a 1-to-1 ratio and then is excreted via the urine or bile [66].

## 4. Role of Ferroptosis in Chronic Diseases

**Figure 2:**
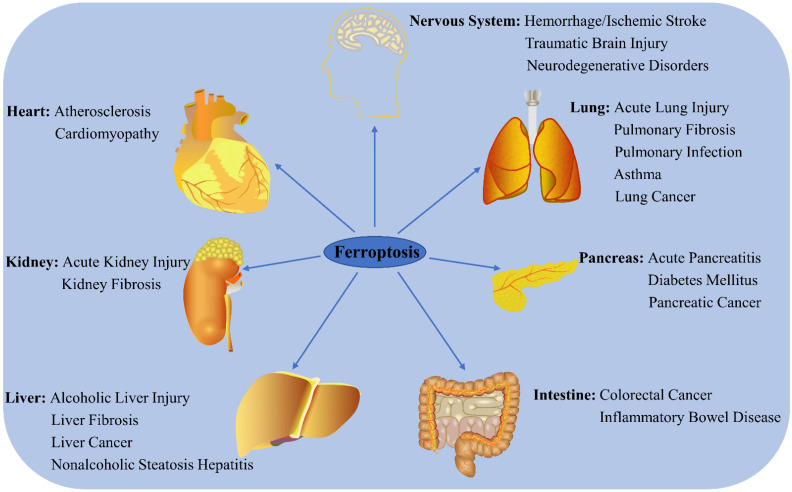
Role of ferroptosis in chronic diseases. Ferroptosis participates in the regulation of many system disorders, including nervous system diseases, cardiovascular diseases, liver diseases, kidney diseases, lung diseases, pancreatic diseases, and intestinal diseases.

Ferroptosis has recently become a hotspot of research focus in the field of disease prognosis and therapy, with numerous studies reporting that it regulates the occurrence and progress of multiple disorders (Figure 2). Here, the latest progress in ferroptosis research and its association with various diseases are summarized.

### 4.1. Cancers

#### 4.1.1. Lung Cancer

Lung cancer is one of the most common causes of cancer-related deaths worldwide. Some key regulators, including KRAS, TP53, Nrf2, YAP, NFS1, STYK1, LSH, RNF113A, and non-coding RNA, are reported to participate in ferroptosis regulation [67]. KRAS mutant lung cancer cells are vulnerable to the ferroptosis induced by SLC7A11 inhibition [68]. P53 also inhibits SLC7A11 expression and cystine uptake, consequently inducing ferroptosis [43]. The activation of Nrf2 negatively regulates ferroptosis by up-regulating various target genes, such as HO-1. Acetaminophen (APAP) sensitizes non-small-cell lung cancer (NSCLC) to erastin-mediated ferroptosis by negatively regulating the Nrf2/HO-1 signaling pathway [69]. Curcumin triggers ferroptosis in NSCLC by activating autophagy [70]. DHA induces lung cancer cell ferroptosis via the inactivation of the PRIM2/SLC7A11 axis [71]. Orlistat promotes lung cancer cell ferroptosis by reducing GPX4 expression and inducing lipid peroxidation [72]. A pure compound extracted from danshen, dihydroisotanshinone I, suppresses the growth of lung cancer cells by triggering both ferroptosis and apoptosis [73]. The artemisinin derivatives artesunate and DHA induce ROS-dependent apoptosis/ferroptosis in NSCLC cells [74]. MiR-27a-3p promotes NSCLC through SLC7A11-mediated-ferroptosis [75], while MiR-302a-3p induces the ferroptosis of NSCLC cells by targeting FPN [76].

#### 4.1.2. Liver Cancer

**Figure 3:**
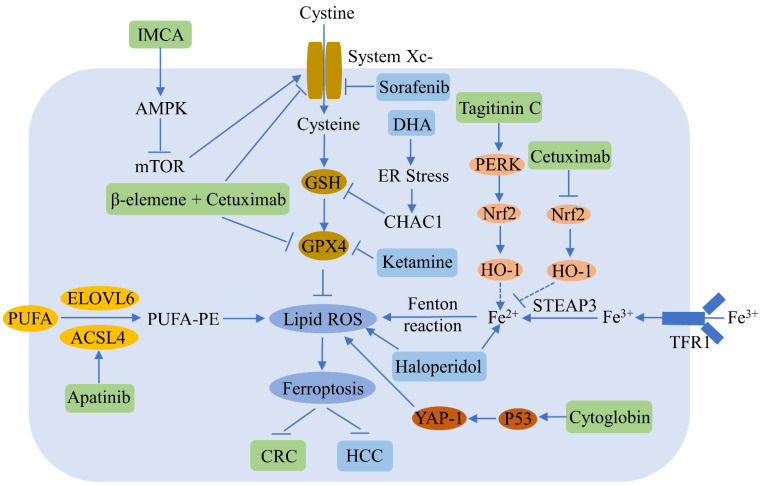
Ferroptosis regulators and pathways in hepatocellular carcinoma (HCC) and colorectal cancer (CRC). Ferroptosis can be a negative regulator of HCC and CRC. Various compounds can inhibit HCC and CRC growth by inducing ferroptosis. The blue and green patterns correspond to regulators for HCC and CRC, respectively. Sorafenib induces HCC ferroptosis by inhibiting SLC7A11. Dihydroartemisinin (DHA) triggers HCC ferroptosis by activating unfolded protein response and upregulating CHAC1. Ketamine increases HCC ferroptosis by inhibiting GPX4. Haloperidol promotes ferroptosis via the increase of Fe^2+^ levels and lipid peroxidation in HCC. IMCA induces CRC ferroptosis by inhibiting SLC7A11. β-elemene and cetuximab combined treatment results in CRC ferroptosis by downregulating GPX4 and SLC7A11. Apatinib enhances CRC ferroptosis by up-regulating ELOVL6/ACSL4 signaling. Tagitinine C induces CRC ferroptosis via the up-regulation of the PERK-Nrf2-HO-1 signaling pathway. Cetuximab increases CRC ferroptosis by downregulating the Nrf2/HO-1 pathway. Cytoglobin increases CRC ferroptosis through the upregulation of p53 and YAP1.

Liver cancer is the sixth most common malignancy and the third primary cause of cancer-related deaths worldwide [77]. Ferroptosis plays an important role in the regulation of hepatocellular carcinoma (HCC) (Figure 3). Sorafenib, which is a multikinase inhibitor used widely in the treatment of advanced HCC, induces ferroptosis by inhibiting SLC7A11; however, activation of the p62-Keap1-Nrf2 pathway suppresses ferroptosis in HCC [78]. Erastin and sorafenib induce ferroptosis by inhibiting Nrf2 expression and activity in HCC [79]. Sorafenib also decreases retinoblastoma protein levels, thereby increasing ferroptosis [80], while the inhibition of metallothionein enhances sorafenib-induced ferroptosis in HCC [81]. Haloperidol strongly promotes erastin- and sorafenib-induced ferroptosis by increasing Fe^2+^ levels and lipid peroxidation in HCC [82]. Ketamine induces ferroptosis via the inhibition of lncRNA PVT1 and GPX4 in liver cancer cells [83]. DHA induces ferroptosis by activating unfolded protein response and upregulating CHAC1 expression in primary liver cancer cells [84]. MiR-214-3p promotes erastin-mediated ferroptosis by decreasing ATF4 expression in hepatoma cells [85]. Artesunate increases the sensitivity of HCC to sorafenib by inducing ferroptosis [86], while O-GlcNAcylation sensitizes liver cancer to RSL3-induced ferroptosis through the YAP/TFRC pathway [87].

#### 4.1.3. Colorectal Cancer

Recent studies have reported that ferroptosis participates in the regulation of colorectal cancer (CRC), a common malignancy of the digestive system (Figure 3). RSL3 is known to induce CRC ferroptosis by inactivating GPX4 and producing ROS [88]. Tagitinine C, a natural product, acts synergistically with erastin to induce ferroptosis in CRC cells via the PERK-Nrf2-HO-1 signaling pathway [48]. The benzopyran derivative IMCA induces ferroptosis by downregulating SLC7A11 and the AMPK/mTOR pathway in CRC [89]. Inhibiting the KIF20A/NUAK1/Nrf2/GPX4 signaling pathway triggers ferroptosis and sensitizes CRC to oxaliplatin [90]. Cetuximab increases the ferroptosis of KRAS mutant CRC, induced by RSL3, by inhibiting the Nrf2/HO-1 signaling pathway [50]. A combined treatment of β-elemene and cetuximab induces KRAS mutant CRC cell ferroptosis via the downregulation of GPX4 and SLC7A11 [91], and the inhibition of SLC7A11 induces the ferroptosis of CRC stem cells [92]. Apatinib enhances ferroptosis in CRC cells by upregulating ELOVL6/ACSL4 signaling [93]. Cytoglobin sensitizes the CRC cells to RSL3 and erastin through the upregulation of p53 and YAP1 [94]. Andrographis enhances the effect of 5-fluorouracil (5FU) against CRC by inducing ferroptosis and inhibiting β-catenin/Wnt-signaling pathways [95]. 

#### 4.1.4. Breast Cancer

Breast cancer is the most common cancer among women. Sulfasalazine has been found to trigger breast cancer cell ferroptosis via the repression of GPX4 and SLC7A11 expressions and an increase in TFR1 and DMT1 expressions, particularly in cells with low estrogen receptor (ER) expression [96]. The combined treatment of siramesine and lapatinib triggers breast cancer cell ferroptosis by increasing TF expression and reducing FPN expression [97]. Metformin triggers ferroptosis via a reduction in the UFMylation of SLC7A11 in breast cancer [98]. Curcumin promotes the ferroptosis of breast cancer cells by increasing lipid ROS levels, lipid peroxidation end-product MDA accumulation, and intracellular free iron levels [99]. Targeted exosome-encapsulated erastin promotes the ferroptosis of triple-negative breast cancer (TNBC) cells [100]. The GSK3β/Nrf2 signaling pathway strengthens the ferroptosis of breast cancer induced by erastin [101]. Inhibiting GPX4 increases gefitinib-induced ferroptosis in TNBC cells [102], while simvastatin induces ferroptosis in TNBC cells [103]. Metformin promotes ferroptosis via the up-regulation of miR-324-3p and down-regulation of GPX4 in breast cancer [104]. Lidocaine induces the ferroptosis of ovarian and breast cancers by increasing miR-382-5p and decreasing SLC7A11 [105], while ketamine induces ferroptosis by targeting the KAT5/GPX4 axis in breast cancer cells [106].

#### 4.1.5. Ovarian Cancer

Ovarian cancers are prevalent female malignancies, seriously affecting women’s health and life quality in the world. Lidocaine promotes ferroptosis in ovarian and breast cancer cells by enhancing miR-382-5p and down-regulating SLC7A11 expression [105]. Stearoyl-CoA desaturase 1 (SCD1) suppresses ovarian cancer cell ferroptosis [107], while the inhibition of pharmaceutical SCD1 promotes ferroptosis in vitro and in vivo, and the combined treatment of SCD1 inhibitors and ferroptosis inducers significantly inhibits ovarian tumor growth [108]. SNAI2 knockdown promotes ferroptosis in ovarian cancer [109]. Sodium molybdate induces the ferroptosis of ovarian cancer cells via labile iron elevation and GSH depletion [110]. Ferroptosis inducers increase the sensitivity of BRCA-proficient ovarian cancer cells to PARP inhibitor by repressing SLC7A11 [25]. Human serum incubated-superparamagnetic iron oxides promote ferroptosis via p53 overexpression in ovarian cancer cells [111]. Superparamagnetic iron oxide nanoparticles increase oxidative stress, reduce autophagy, and activate ferroptosis in ovarian cancer stem cells [111]. GALNT14 induces ferroptosis in ovarian cancer via the EGFR/mTOR pathway [112]. MAP30 protein from *Momordica charantia* and cisplatin synergistically induce ferroptosis in ovarian cancer [113].

#### 4.1.6. Pancreatic Cancer

Pancreatic cancer, one of the most fatal of all cancers, is seen mainly in males and the older population (40–85 years) [114]. The inhibition of cytosolic aspartate aminotransaminase promotes pancreatic cancer cell ferroptosis by repressing mitochondrial metabolism and promoting a catabolic state [115]. The natural compound artesunate induces ferroptosis in pancreatic cancer cells [116]. Cysteine depletion promotes the ferroptosis of pancreatic tumor cells in mice [117]. Ruscogenin, a saponin found in the root of *Ophiopogon japonicus*, increases intracellular ferrous iron and ROS, thereby inducing ferroptosis in pancreatic cancer cells [118]. The abrogation of ADP ribosylation factor 6 promotes RSL3-induced ferroptosis in pancreatic cancer cells [119]. DHA acts synergistically with cisplatin to trigger ferroptosis in pancreatic ductal adenocarcinoma (PDAC) by regulating iron metabolism [120]. Combining artesunate with GRP78 inhibition facilitates ferroptosis in KRAS mutant PDAC [121]. Chrysin enhances pancreatic cancer sensitivity to gemcitabine by inducing autophagy-dependent ferroptosis via the targeting of human carbonyl reductase 1 [122]. Ponicidin suppresses pancreatic cancer growth by inducing ferroptosis [123], while piperlongumine induces ferroptosis in human pancreatic cancer cells by suppressing the gamma-glutamyl cycle and regulating the metabolism of PUFAs [124].

### 4.2. Nervous System Diseases

#### 4.2.1. Stroke

Stroke is one of the leading causes of death and disability in the world. Selenium activates homeostatic transcription to protect neuron cells from ferroptosis and treat stroke [125]. ACSL4 enhances ischemic stroke by inducing neuronal ferroptosis-related brain injury and neuroinflammation [126], while the inhibition of ACSL4 improves neurological functioning after stroke via the suppression of ferroptosis [127]. Reducing NCOA4 inhibits the ferritinophagy-mediated ferroptosis of neurons, and thus can be used to treat ischemic stroke [63]. The hemin-induced hemorrhagic stroke model is one of classic neuronal ferroptosis [128]. Intracerebral hemorrhage (ICH) is a type of severe stroke, the pathology of which is closely related to ferroptosis. Curcumin nanoparticles inhibit ferroptosis and enhance ICH treatment [129], and baicalin can also suppress ferroptosis in ICH [130]. Tau-mediated iron export inhibits ferroptosis after ischemic stroke [131]. Supplementing lactoferrin reduces the ferroptosis of nerve cells after diabetic ICH [132], while crocin reduces ICH-induced neuronal ferroptosis by increasing Nrf2 expression and nuclear translocation [133]. Dauricine represses nerve cell ferroptosis and brain injury after ICH by up-regulating GPX4 expression [134], while pyridoxal isonicotinoyl hydrazone, a lipophilic iron-chelating agent, alleviates hemorrhage stroke by decreasing ferroptosis and inflammation [135]. 

#### 4.2.2. Traumatic Brain Injury

Traumatic brain injuries (TBIs) occur worldwide and result in serious economic burden. Inhibiting ferroptosis reduces tissue damage and ameliorates long-term motor and cognitive function after TBI in mice [136]. Biomarkers of ferroptosis are increased after TBI; however, baicalein decreases neuronal ferroptosis and improves post-TBI outcome [137]. Ferritin H deletion in nerve cells abolishes the neuroprotection of melatonin against TBI-mediated ferroptosis [138]. Prokineticin-2 prevented neuronal cells from ferroptosis in a TBI model [139]. Polydatin alleviates TBI via the inhibition of ferroptosis [140]. MiR-212-5p attenuates neuronal ferroptosis after TBI by targeting PTGS2 [141]. Ruxolitinib was found to protect against neuronal ferroptosis in a mouse TBI model [142]. Ferristatin II, an iron uptake inhibitor, prevents neuronal ferroptosis after TBI [143], while tetrandrine ameliorates TBI by regulating autophagy to reduce ferroptosis [144]. 

#### 4.2.3. Alzheimer’s Disease

Alzheimer’s disease (AD) is the most ordinary type of dementia, featuring β-amyloid and Tau protein accumulation and aggregation [145]. FPN loss induces ferroptosis and promotes memory impairment in AD [146]. NADPH oxidase 4 promotes astrocytes ferroptosis via oxidative-stress-induced lipid peroxidation in AD [147]. Mitochondrial aldehyde dehydrogenase reduces AD-induced cardiac anomalies by inhibiting ACSL4-mediated ferroptosis [148]. Tetrahydroxy stilbene glycoside was shown to improve AD in APP/PS1 mice via the inhibition of GPX-related ferroptosis [149], while eriodictyol improves cognitive disorder in APP/PS1 mice by suppressing ferroptosis via Nrf2 activation mediated by a vitamin D receptor [150]. In addition, forsythoside A alleviates AD by reducing Nrf2/GPX4 activation-induced ferroptosis [151].

#### 4.2.4. Parkinson’s Disease

Parkinson’s disease (PD) is a common neurodegenerative disorder characterized by motor impairment. FTH1 was reported to suppress ferroptosis by impairing ferritinophagy in a 6-OHDA-induced PD model [152]. Activating the p62-Keap1-Nrf2 pathway inhibits the ferroptosis triggered by 6-OHDA in dopaminergic cells [153]. Thioredoxin-1 reduces ferroptosis in PD by up-regulating GPX4 and GSH [154]. Super-enhancer-driven sorting nexin 5 expression facilitated the ferroptosis of dopaminergic neurons in PD models [155]. MiR-335 enhanced ferroptosis by degrading FTH1 in in vitro and in vivo models of PD [156]. Moxibustion presents a neuroprotective function via anti-ferroptosis in PD [157,158]. Ferritinophagy-mediated ferroptosis involves in paraquat-induced neurotoxicity in PD [159]. 

#### 4.2.5. Huntington’s Disease

Huntington’s disease (HD) is an autosomal dominant and fatal neurodegenerative disease resulting from abnormal cytosine-adenine-guanine repetition in the huntingtin gene [160], for which there is currently no effective treatment. A few ferroptotic characteristics have been observed in HD animal models and patients, such as iron accumulation, oxidative stress, GSH depletion, and reduced GPX activity [161], which suggests the participation of ferroptosis in the regulation of HD pathogenesis. Several ferroptosis regulators have also been found to take effect in HD models. For instance, Fer-1 suppressed oxidative lipid damage and ferroptosis in HD cellular models [162], while the intraventricular delivery of the iron chelator DFO led to motor phenotype improvement in R6/2 HD mice [163]. 

### 4.3. Metabolic Diseases

#### 4.3.1. Cardiovascular Diseases

Cardiovascular diseases are those involving the heart and blood vessels, such as atherosclerosis, peripheral vascular diseases, and cerebrovascular diseases. Ferritin plays a key role in inhibiting cardiac ferroptosis and succedent heart failure, while cardiac ferritin H loss promotes cardiomyopathy by increasing SLC7A11-mediated ferroptosis [23]. Mitochondria-dependent ferroptosis plays an important role in doxorubicin-induced cardiomyopathy [164]. ENPP2, a lipid kinase participating in lipid metabolism, reduces erastin-induced ferroptosis in cardiomyocytes by regulating GPX4, ACSL4, and Nrf2 expression and increasing AKT signaling [165]. The inhibition of ferroptosis alleviates atherosclerosis by reducing lipid peroxidation and endothelial dysfunction [9]. Rapamycin plays a key role in reducing excess iron and ferroptosis in cardiomyocytes [166]. MSC exosomes derived from human umbilical cord blood inhibit ferroptosis and attenuate myocardial injury, possibly by inhibiting the expression of DMT1 by miR-23a-3p [167]. GPX4 down-regulation during myocardial infarction results in ferroptosis in cardiomyocytes [32]. TRIM21 ablation alleviates cardiotoxicity of the chemotherapeutic agent doxorubicin by suppressing ferroptosis [168].

#### 4.3.2. Diabetes Mellitus

Diabetes mellitus is classified as either type 1 diabetes mellitus (T1DM) or type 2 diabetes mellitus (T2DM), the latter of which affects approximately 90% to 95% of all diabetes mellitus patients. Quercetin potentially alleviates T2DM by decreasing pancreatic iron deposition and pancreatic β cell ferroptosis [169]. SLC40A1 mediates ferroptosis and cognitive dysfunction in T1DM [170]. SIRT3 deficiency suppresses autophagy-mediated ferroptosis by suppressing AMPK-mTOR pathway activation and enhancing GPX4 levels, thus providing a potential therapeutic approach for gestational diabetes mellitus [34]. Melatonin reduces ferroptosis levels by activating the Nrf2/HO-1 signaling pathway in T2DM osteoporosis [55]. Ferroptosis participates in the development of diabetic nephropathy (DN); however, Nrf2 upregulation inhibits ferroptosis and delays the progression of DN [171]. Ferroptosis might enhance diabetic renal tubular injury via the HIF-1α/HO-1 pathway [172]. HMOX1 upregulation enhances ferroptosis in diabetic atherosclerosis [173]. Autophagy inhibition leads to the ferroptosis of cardiomyocytes and worsens diabetic cardiomyopathy development in mice by targeting Nrf2 [174]. DFO treatment alleviates poststroke cognitive impairment in diabetes by inhibiting ferroptosis [175]. Caveolin-1 reduces diabetes-related cognitive disorder by regulating neuronal ferroptosis-mediated mitochondrial homeostasis [176].

#### 4.3.3. Liver Diseases

Increasing evidence indicates that the suppression of ferroptosis may slow the development of several liver diseases, including alcoholic liver injury, nonalcoholic steatosis hepatitis (NASH), and fibrosis [177]. Intestinal SIRT1 deficiency attenuates alcoholic liver injury via the mitigation of hepatic ferroptosis in mice [178]. Ginkgolide B, a primary ingredient of *Ginkgo biloba* extracts, alleviates nonalcoholic fatty liver disease in obese mice by inhibiting ferroptosis, possibly through the Nrf2 signaling pathway [179]. Ferroptosis inhibitors reduce methionine/choline-deficient diet-induced NASH by inhibiting liver lipotoxicity [180]. (+)-Clausenamide, an active alkaloid isolated from the leaves of *Clausena lansium* (Lour.) Skeels, alleviates drug-induced liver injury by suppressing hepatocyte ferroptosis. The radical oxidation of n-6 PUFAs promotes ferroptosis and APAP-induced acute liver failure [181]. Ferroptosis plays a dual role in liver fibrosis. Some evidence suggests the pathogenic role of ferroptosis in iron-overload-induced liver fibrosis, and inhibiting ferroptosis potently prevents liver fibrosis. Hepatic TF plays a role in maintaining liver function and preventing ferroptosis-induced liver fibrosis [182]. Artesunate relieves liver fibrosis via downregulation of the ferroptosis signaling pathway [183]. Fibroblast growth factor 21 reduces iron-overload-induced liver injury and fibrosis by suppressing ferroptosis [184]. Activation of hepatic stellate cells (HSCs); that is, transdifferentiation into matrix-producing myofibroblasts, is considered the central driver of hepatic fibrosis [185]. Recent studies demonstrate the potential of inducing ferroptosis in HSCs as a therapeutic strategy designed to alleviate the development of liver fibrosis. Artemether relieves carbon-tetrachloride-induced liver fibrosis and inhibits HSCs activation through p53-dependent ferroptosis induction [186]. Sorafenib suppresses liver fibrosis by inducing HSCs ferroptosis via the inactivation of the HIF-1α/SLC7A11 pathway [187]. Moreover, certain regulators of ferroptosis in HSCs, including p53 [186], ELAV-like protein 1 (ELAVL1) [188], and zinc finger protein 36 (ZFP36) [189], have been reported as potential targets for the treatment of liver fibrosis.

#### 4.3.4. Kidney Diseases

Ferroptosis has recently been associated with diverse kidney diseases, including acute kidney injury (AKI) and polycystic kidney disease. Quercetin reduces AKI by suppressing ferroptosis [190], while nuciferine also reduces folic-acid-induced AKI via the suppression of ferroptosis [191], and the inactivation of ferroptosis regulator GPX4 results in AKI in mice [37]. Inhibiting ferroptosis attenuates AKI in rats with severe acute pancreatitis [192], while the silencing of miR-182-5p and miR-378a-3p reduces ischemia/reperfusion-induced renal injury in rats by suppressing ferroptosis [193]. The inhibition of ferroptosis by XJB-5-131 reduces renal tubular cell injury in kidney diseases [194]. Targeted inhibition of the circadian clock components Rev-erb-α/β suppresses ferroptosis to reduce folic acid-induced AKI [195]. Dimethyl fumarate inhibits ferroptosis to attenuate AKI by targeting Nrf2 [196]. Fenofibrate inhibits ferroptosis by upregulating Nrf2, thereby resisting DN progression [171]. Activating the vitamin D receptor attenuates cisplatin-induced AKI by repressing ferroptosis partially through GPX4 trans-regulation [197]. Legumain enhances tubular ferroptosis via the activation of chaperone-mediated GPX4 autophagy in AKI [27]. Tocilizumab mimotopes reduce kidney injury and fibrosis by blocking IL-6 signaling and ferroptosis [198]. ACSL4 deficiency alleviates ferroptosis-mediated AKI [199].

#### 4.3.5. Lung Diseases

Recent studies have suggested that ferroptosis plays an important role in a number of lung diseases, including acute lung injury (ALI), chronic obstructive pulmonary disease, pulmonary fibrosis, pulmonary infection, and asthma. Fer-1 relieves LPS-induced ALI by suppressing ferroptosis [200]. Nrf2 inhibits ferroptosis and alleviates ALI by increasing SLC7A11 and HO-1 expression [24]. The inhibitor of apoptosis-stimulating protein of p53 reduces IIR-ALI via the suppression of ferroptosis [201]. Panaxydol attenuates LPS-induced ALI by up-regulating the Keap1-Nrf2/HO-1 pathway to inhibit ferroptosis [56]. Wedelolactone mitigates acute pancreatitis associated with lung injury by increasing GPX4 to suppress pyroptosis and ferroptosis [202]. Nrf2 alleviates seawater-drowning-induced ALI by suppressing ferroptosis [203]. Nrf2 and STAT3 reduce IIR-ALI by decreasing SLC7A11-mediated ferroptosis [26]. Melatonin reduces PM2.5-induced lung injury through the suppression of lung epithelial cell ferroptosis via Nrf2 activation [204]. Sevoflurane reduces LPS-induced ALI by repressing ferroptosis via the upregulation of HO-1 expression [205]. Obacunone reduces LPS-induced ALI by suppressing ferroptosis via the increase of Nrf2-dependent antioxidant responses [206]. Hydrogen sulfide reduces ferroptosis and activates autophagy by downregulating mTOR signaling in sepsis-induced ALI [207]. The inhibition of ACSL4 mitigates ferroptosis in ischemia/reperfusion-induced lung injury by decreasing lipid peroxidation and increasing the GSH and GPX4 levels [208].

### 4.4. Inflammatory Bowel Diseases

Inflammatory bowel disease is a chronic relapsing disease mainly affecting the intestinal tract, and includes ulcerative colitis (UC) and Crohn’s disease (CD). Ferroptosis participates in intestinal epithelial cell death in UC [209]. The suppression of ferroptosis ameliorates DSS-induced UC by suppressing the Nrf2/HO-1 signaling pathway [49]. Curculigoside protects against ferroptosis in UC by inducing GPX4 [210]. Fer-1 reduces TNBS-induced colitis by inhibiting ferroptosis [211]. Furin inhibits the DSS-induced ferroptosis of epithelial cells and reduces experimental colitis via Nrf2-GPX4 signaling pathway activation [212]. Astragalus polysaccharide was shown to inhibit ferroptosis in a murine model of experimental colitis and human Caco-2 cells by blocking the Nrf2/HO-1 pathway [213]. Dietary lipids are a trigger of GPX4-restricted enteritis resembling CD [214]. 

## 5. Regulatory Effects of Food-Borne Active Ingredients on Ferroptosis

**Figure 4:**
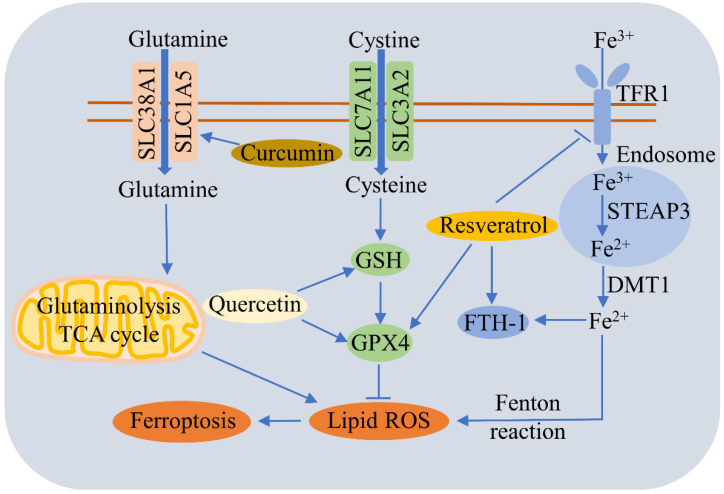
Regulatory effects of food-borne active ingredients on ferroptosis. This schematic diagram shows the regulation pathways of ferroptosis by polyphenols such as quercetin, curcumin, and resveratrol. Curcumin triggers ferroptosis by promoting SLC1A5-mediated glutamine uptake. Quercetin alleviates ferroptosis by upregulating GSH and GPX4. Resveratrol reduces ferroptosis by decreasing TfR1 expression and increasing the expressions of FTH1 and GPX4.

A large number of studies have reported that food-borne active ingredients, such as polyphenols, can regulate ferroptosis (Figure 4). 

Quercetin (QCT), a natural flavonoid found commonly in fruits and vegetables, alleviates T2DM by inhibiting the ferroptosis of pancreatic β cells via the upregulation of GSH and GPX4, and by increasing mitochondria membrane-associated protein VDAC2, which functions as an antioxidant [169]. QCT also reduces AKI by repressing ferroptosis via a reduction in MDA and lipid ROS levels and an increase in GSH [190]. QCT can suppress the erastin-induced ferroptosis of bone-marrow-derived mesenchymal stem cells, probably via the antioxidant pathway [215]. QCT triggers p53-mediated cancer cell ferroptosis by promoting lysosome-dependent ferritin degradation and ROS generation [216]. Dihydroquercetin reverses cigarette-smoke-induced ferroptosis in the pathogenesis of chronic obstructive pulmonary disease by up-regulating the Nrf2-dependent pathway [217]. 

Gallic acid (GA) is a natural polyhydroxy phenolic compound seen in various foods, such as edible mushrooms, fruits, and vegetables. GA triggers cancer cell death via the activation of apoptotic, ferroptotic, and necroptotic pathways [218]. Preirradiation therapy followed by GA treatment inhibits the survival of cancer cells more effectively than GA treatment alone via the apoptosis and ferroptosis cell death mechanisms [219].

Curcumin, a polyphenol compound extracted from the turmeric plant, enhances the treatment effect of NSCLC by activating autophagy-dependent ferroptosis [70]. Curcumin decreases rhabdomyolysis-related renal damage by reducing ferroptosis, and mechanistic studies have shown that curcumin downregulates the TLR4/NF-κB axis and activates HO-1 [220]. Since curcumin nanoparticles suppress ferroptosis, they can be used to strengthen the treatment of ICH [129]. Curcumin also induces the ferroptosis of breast cancer cells by upregulating SLC1A5 [99].

Epigallocatechin gallate (EGCG), a major polyphenol in green tea, protects against radiation-induced intestinal injury by scavenging ROS and repressing apoptosis and ferroptosis via the Nrf2 signal pathway [221]. EGCG pretreatment reduces doxorubicin cardiotoxicity-induced ferroptosis by increasing AMPKα2 and promoting adaptive autophagy [222]. Apigenin is a flavonoid found in green leafy herbs and vegetables, including celery, parsley, spinach, chamomile, green pepper, and eggplant, as well as oranges and red wine [223]. Apigenin is able to alleviate myeloperoxidase-mediated oxidative stress and repress ferroptosis in neuronal cells [224].

Resveratrol is a polyphenol that exists commonly in various vegetables and fruits, such as grapes. Resveratrol alleviates ferroptosis-induced myocardial ischemia/reperfusion injury, reduces TfR1 expression, and increases the expressions of FTH1 and GPX4 [225]. Resveratrol nanoparticles can repress erastin-induced ferroptosis in HT22 mouse hippocampal cells [226], and also inhibits acrolein-induced ferroptosis and insulin secretion disorder via the ER-stress-related PERK pathway in mouse pancreatic β cells [227]. Nobiletin, a critical active flavonoid in citrus fruits, was found to reduce ferroptosis-related renal injury, inflammation, and fibrosis in a unilateral ureteral obstruction mouse model [228]. Nobiletin triggers the ferroptosis of human skin melanoma cells via the GSK3β-mediated Keap1/Nrf2/HO-1 signaling pathway [229].

## 6. Conclusions

Ferroptosis is a recently identified type of programmed cell death that results from iron-dependent lipid peroxide accumulation. Scientific research developments have revealed the participation of ferroptosis in the pathophysiological processes of numerous diseases, creating the potential for novel approaches to treat these diseases. The induction of ferroptosis can inhibit tumor progression, while its inhibition can alleviate nervous system diseases, metabolic diseases, and inflammatory bowel diseases. A large number of studies have reported that food-borne active ingredients, such as polyphenols, can regulate ferroptosis, either inhibiting it and thereby alleviating chronic conditions, or triggering ferroptosis in cancer cells and thereby inhibiting cancer. To define more precise relationships between ferroptosis and health effects, the following issues need to be addressed in the future:

- The relationship between ferroptosis and other types of cell death still needs to be defined;
- The final executors of ferroptosis downstream of lipid peroxidation remain to be identified;
- In addition to polyphenols, other food-borne active ingredients that regulate iron death remain to be investigated.

## Figures and Tables

**Table 1:** Characteristics of ferroptosis, apoptosis, autophagy, necroptosis and pyroptosis.

	Ferroptosis	Apoptosis	Autophagy	Necroptosis	Pyroptosis
Morphological features	Mitochondrial shrinkage with increased mitochondrial membrane densities, reduced or vanished mitochondria crista, rupture of outer mitochondrial membrane	Plasma membrane blebbing, cellular and nuclear volume reduction, rounding-up of the cell, nuclear fragmentation, chromatin condensation	Formation of double-membraned autolysosomes	Rupture of plasma membrane, generalized swelling of the cytoplasm and organelles, moderate chromatin condensation	Karyopyknosis, cell edema and membrane rupture
Biochemical features	Iron accumulation and lipid peroxidation	DNA fragmentation	Increased lysosomal activity	Drop in ATP levels	Dependent on caspase-1 and proinflammatory cytokine releases
Genetic features	Positive: PTGS2, ACSL4, TFR1, NCOA4; Negative: GPX4, SLC7A11, NRF2, FSP1, FTH1	Positive: Bax, Bak, Bad, Bim, Bid; Negative: Bcl-2, Bcl-XL, Mcl-1	Positive: ATG5, ATG7, LC3, Beclin-1	Positive: RIP1, RIP3, MLKL	Positive: Caspase-1, IL-1β, IL-18

## Data Availability

Not applicable.
